# Gene co-expression network analysis in zebrafish reveals chemical class specific modules

**DOI:** 10.1186/s12864-021-07940-4

**Published:** 2021-09-13

**Authors:** Prarthana Shankar, Ryan S. McClure, Katrina M. Waters, Robyn L. Tanguay

**Affiliations:** 1 Department of Environmental and Molecular Toxicology, Sinnhuber Aquatic Research Laboratory, 28645 East Highway 34, Oregon State University, Corvallis, OR 97331 USA; 2 Biological Sciences Division, Pacific National Northwest Laboratory, 902 Battelle Boulevard, P.O. Box 999, Richland, WA 99352 USA

**Keywords:** Zebrafish, Gene co-expression, Transcriptomics, Aryl hydrocarbon receptor, Flame retardant chemicals, Network, Development

## Abstract

**Background:**

Zebrafish is a popular animal model used for high-throughput screening of chemical hazards, however, investigations of transcriptomic mechanisms of toxicity are still needed. Here, our goal was to identify genes and biological pathways that Aryl Hydrocarbon Receptor 2 (AHR2) Activators and flame retardant chemicals (FRCs) alter in developing zebrafish. Taking advantage of a compendium of phenotypically-anchored RNA sequencing data collected from 48-h post fertilization (hpf) zebrafish, we inferred a co-expression network that grouped genes based on their transcriptional response.

**Results:**

Genes responding to the FRCs and AHR2 Activators localized to distinct regions of the network, with FRCs inducing a broader response related to neurobehavior. AHR2 Activators centered in one region related to chemical stress responses. We also discovered several highly co-expressed genes in this module, including *cyp1a*, and we subsequently show that these genes are definitively within the AHR2 signaling pathway. Systematic removal of the two chemical types from the data, and analysis of network changes identified neurogenesis associated with FRCs, and regulation of vascular development associated with both chemical classes. We also identified highly connected genes responding specifically to each class that are potential biomarkers of exposure.

**Conclusions:**

Overall, we created the first zebrafish chemical-specific gene co-expression network illuminating how chemicals alter the transcriptome relative to each other. In addition to our conclusions regarding FRCs and AHR2 Activators, our network can be leveraged by other studies investigating chemical mechanisms of toxicity.

**Supplementary Information:**

The online version contains supplementary material available at 10.1186/s12864-021-07940-4.

## Background

With advancements in technology and medical science, various types of chemicals (xenobiotics, drugs, etc) are being applied to both the natural environment and human body. However, the majority of these chemicals are yet to be evaluated for their potential to cause adverse health effects. High-throughput (HTP) in vitro assays are popular methods used to estimate chemical toxicity and identify underlying molecular events [1]. Despite their application to determine whether a chemical class may be toxic, we still lack adequate knowledge of the mechanisms of toxicity of many chemicals [2] preventing us from connecting assay measurements to phenotypes. Animal models can be a more translatable way of revealing chemical hazard potential to humans, with both metabolism and integrated tissue systems. However, high cost and low throughput are often barriers to testing across a large chemical space. These barriers have been serious deficiencies in advancing chemical risk assessment, and thus a HTP and accurate prediction of which chemicals may be toxic is an essential next step in the evaluation of chemical safety [3].

A popular HTP animal model, zebrafish (*D. rerio*), by virtue of its rapid development [4] and high physiological and genetic similarity to humans [5], is being leveraged to identify chemical hazards [6] and to determine the molecular signaling events that precede adverse phenotypic outcomes [7]. One of the main -omics techniques that is applied to zebrafish is transcriptomics, which uses RNA sequencing analysis to provide an unbiased snapshot of gene expression changes associated with a particular chemical exposure [8]. Numerous transcriptomic studies in both developing and adult zebrafish leave no doubt that diverse chemical exposures often result in diverse gene expression changes [9–11]. Such studies have led to the discovery of transcripts important for the manifestation of higher level toxicity effects [12, 13] and to a large relational database of chemical-zebrafish transcriptome responses.

Much of this transcriptomic data collected from developing zebrafish has examined toxicants from a variety of different classes. Polycyclic aromatic hydrocarbons (PAHs), are a large class of ubiquitous environmental pollutants, and human exposure has been linked to cardiovascular disease, respiratory problems, carcinogenicity, and developmental deficits [14, 15]. The molecular initiating event of many PAHs is the aryl hydrocarbon receptor (AHR), and PAHs have been shown to bind all three orthologs of AHR in zebrafish, with AHR2 being the predominant receptor required for toxicity [16]. 2,3,7,8-Tetrachlorodibenzodioxin (TCDD) is a halogenated aromatic hydrocarbon and the best-characterized and most potent AHR2 ligand. It is widely used as a canonical xenobiotic ligand to study responses downstream of AHR activation [17]. Many studies have focused on the roles of the cytochrome P450 (CYP) metabolizing enzymes in PAH toxicity [18, 19], while other molecular signaling events downstream of the AHR remain largely unknown. Flame-retardant chemicals (FRCs) are a diverse class of chemicals including polybrominated diphenyl ethers (PBDEs) and organophosphate flame retardants (OPFRs), commonly applied in an additive manner to manufactured materials such as furniture, clothing, and electronics, and often found to leach into surrounding environments and human bodies [20, 21]. FRCs have been associated with neurodevelopmental effects [22], altered reproductive and thyroid function [23], and impacts on the immune and endocrine systems [24]. While many research groups have conducted transcriptomic studies in developing zebrafish exposed to PAHs and TCDD, only a limited number of FRC whole-genome expression studies have been published [25–28].

The standard use of transcriptomic data, and the approach used in many of the studies above, is to compare gene expression levels between a control and a chemical treatment. However, with a transcriptomic database of sufficient size [29], it is possible to perform a meta-analysis of sequencing data and construct a network of genes based on co-expression values of each gene pair [30, 31]. Co-expressed genes show a similar pattern of either direct or inverse co-expression across multiple conditions or biological replicates. Network analysis can be used to reveal important chemical targets in a biological system [32–34], and to indicate processes that are critical for or distinctive between responses to certain classes of chemicals [35, 36]. Network approaches also have a distinct advantage over standard control/condition comparisons in that data from several different studies, even those conducted under variable conditions, can be collected and integrated to produce a model formed from a compendium of many datasets. Network analysis can also highlight genes, pathways, and processes that may change their expression in a significant but subtle manner (less than the 2-fold cutoff normally applied to control/condition comparisons), expanding our ability to identify processes related to chemical class or phenotypic response. A whole transcriptome approach is thus far more efficient and informative to detect subtle but potentially important shifts in gene expression patterns that describe interactions between processes impacted by chemical exposure [37]. Such patterns would be missed by smaller scale targeted expression studies for specific biomarkers. Based on the large amount of transcriptomic data collected for zebrafish exposed to a variety of different chemicals, we are now able to apply these network approaches to zebrafish response to chemicals.

While phenotypic outcomes of many chemicals have been studied in zebrafish [6, 38], we still do not know many of the transcriptomic pathways and processes that are induced by these chemicals early in response. Additionally, we do not know to what degree different classes of chemicals may overlap in their transcriptomic response compared to their potentially similar phenotypic responses. To fill this gap in knowledge, the primary goal of our study was to utilize network analyses to discover both common and distinctive biological pathways that polycyclic aromatic hydrocarbons (PAHs), 2,3,7,8-Tetrachlorodibenzodioxin (TCDD), and several flame retardant chemicals (FRCs), alter in developing zebrafish. These chemicals were selected in part because transcriptomic data reflecting a similar experiment design was available. However, these treatments not only encompassed chemicals with unknown and likely distinct modes of action, but also included those with the same or similar modes of action, allowing us to compare the individually altered transcriptomes in a single network. Networks were inferred using a random forest method [31] applied to the compendium of both new and previously published zebrafish transcriptomic data. We identified specific portions of this network that represented tightly co-expressed genes enriched for certain pathways and responding to certain chemical types. The network approach used here enabled discovery of novel genes in the AHR2 signaling pathway as well as several genes and pathways associated with FRC exposure. The inference of this gene co-expression network for zebrafish not only provides novel insight into the transcriptional responses to chemical exposure, but can serve as a resource for other studies focused on transcriptomic coordination, predictive toxicology, and identification of chemical-specific biomarkers and processes of interest in this model organism.

## Results

### Characterization of full dataset

**Fig. 1:**
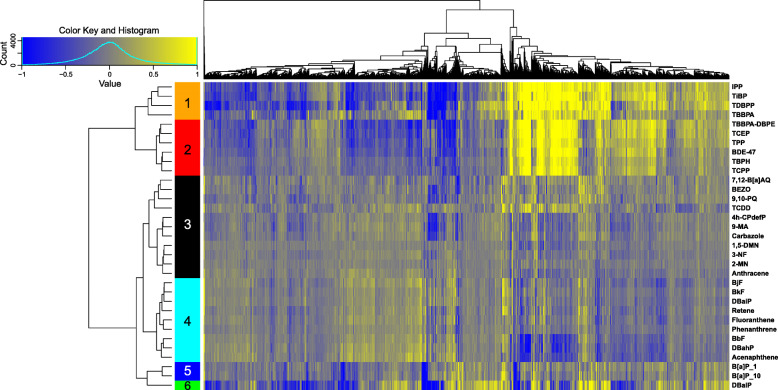
Heatmap of 48-hpf zebrafish transcriptomic response to ten Flame Retardant Chemical (FRC), 22 Polycyclic Aromatic Hydrocarbon (PAH), and 1 TCDD treatment. Average log2 fold change (log2FC) values for each chemical treatment (y-axis) are shown, with yellow indicating higher expression and blue indicating lower expression compared to each chemical’s DMSO control. Both genes and treatments are grouped via hierarchical clustering with clusters of treatments (Clusters 1–6) indicated by colors on the left

In this study, we sought to identify molecular signatures and biological pathways following exposures to a diverse group of chemicals. This was done using a large array of transcriptomic data combined with the first chemical-focused gene co-expression network inferred using zebrafish. Transcriptomic data from 48-h post fertilization (hpf) zebrafish included several previously published studies [7, 39–41] in addition to new unpublished datasets. Details on these datasets including the exposure concentrations and the number of differentially expressed genes (DEGs) are included in Supplementary Table S1. To gain an overview of transcriptomic patterns from the 33 unique chemical treatments, we first used Ward’s method of hierarchical clustering of the 10,346 genes that responded significantly to at least a subset of chemicals. We observed that the treatments naturally clustered into six groups based on log_2_FC values (Fig. 1). Clusters 1 (orange) and 2 (red) represented all the flame retardant chemicals (FRCs) examined and were also more similar to each other than to other clusters in the dataset, showing that the overall transcriptomic response to FRCs is distinct from PAH and TCDD responses. In addition to differences in transcriptomic response based on chemical type, we also found that when we compared our clustering to our lab’s previous studies investigating the morphology and behavior effects caused by FRC exposure [6, 41], there were differences between transcriptomic response and developmental toxicity phenotype. For example, IPP exposure caused both morphology and behavior malformations at 120 hpf and yet, it clustered here with the relatively benign FRCs. While several PAHs in Cluster 3 (black) (9-MA, 3-NF, 2-MN, 1,5-DMN, Carbazole, and Anthracene) were previously determined to cause modest or no phenotypic responses in developing zebrafish, other chemicals in this cluster including 4 h-CPdefP, 7,12-B [a] AQ, and TCDD are known to cause overt developmental toxicity [7, 38, 42]. These observations demonstrated that transcriptional response and phenotypic outcome are often not strongly correlated. Clusters 4 and 5 consisted of the remaining PAHs in this study. We noted that within all the PAHs and TCDD, the chemicals known to predominantly activate AHR2 (“AHR Activators”, see Supplementary Table S1) did not group together. The hierarchical clustering analysis presented here indicates that chemical type is the strongest driver of overall transcriptomic response, rather than known mechanism of action or magnitude of developmental toxicity within the chemical classes.

### Global analysis of full co-expression network

**Fig. 2:**
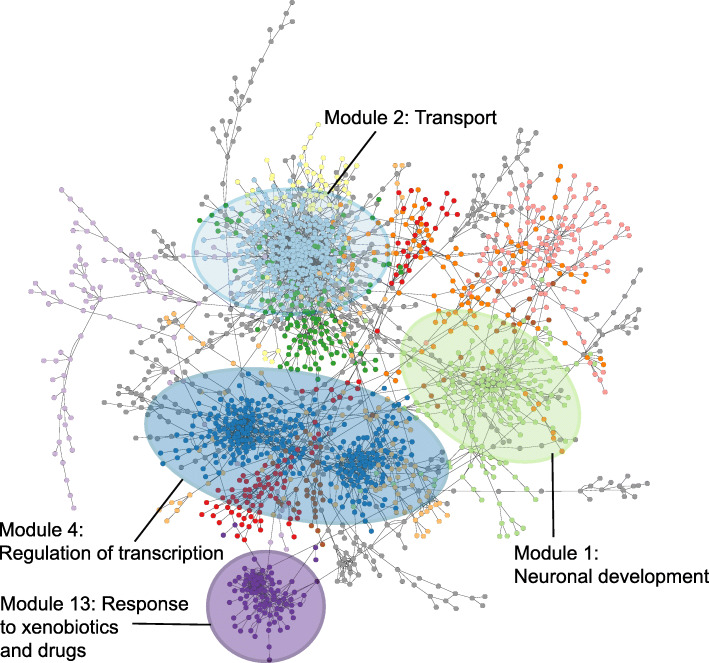
Gene co-expression network of 48-hpf zebrafish transcriptomic response to chemicals. Small colored circles represent zebrafish genes (nodes), and lines (edges) connecting the genes represent instances of high co-expression. Nodes are colored by the module they belong to with processes highly enriched in four example modules indicated

We next inferred a co-expression network using the random forest method, GENIE3 [31], and grouped genes into one of 23 different modules ranging in size from 14 to 425 genes. We chose to use GENIE3 as we have found it be highly accurate in our previous work [43], as well as in other studies that directly compare gene co-expression network methods [29]. While these previous studies were in bacterial or human systems, we used similar methods like those we already published [43] to compare GENIE3 to other gene co-expression methods using the zebrafish data in this study. Again, GENIE3 was found to be the most accurate and provided the most comprehensive network with our data (data not shown). Figure 2 shows the location of the 12 largest modules calculated based on the number of genes they each contain. Table 1 provides module information including number of genes in each module and colors for each module used in Fig. 2. We carried out functional enrichment on all modules using g:Profiler [44] and found that several modules were significantly enriched (adjusted *p-value* < 0.05, function is overrepresented in a module compared to the whole genome) for one or more processes (Supplementary Table S2). Table 1 shows the pathway(s) that had the highest functional enrichments for each module, and demonstrates the diversity in functional enrichment across all the modules, with some examples of the most enriched functions highlighted in Fig. 2. Many of the functions are related to disruptions to normal development such as nervous system (Modules 1 and 5) and axon development (Module 10), in addition to processes associated with ion transporter activities (Modules 2 and 6) and gene expression (Modules 4 and 14).

**Table 1:**
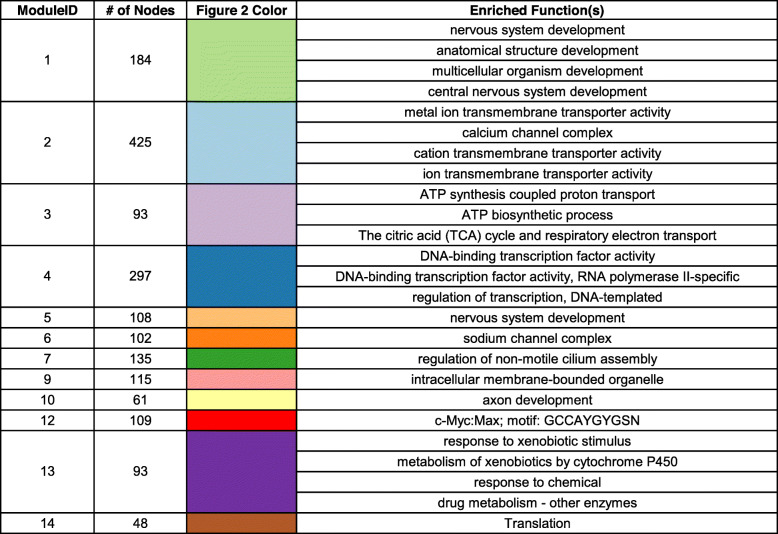
Top GO term enrichment of genes in 12 largest modules of the full gene co-expression network

**Fig. 3:**
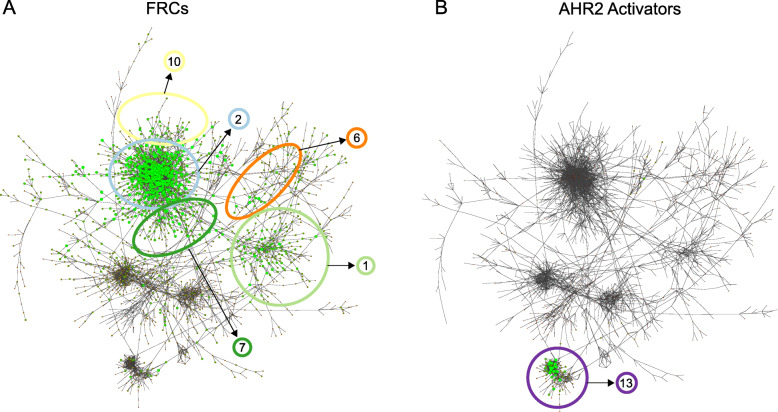
Network response to FRCs and AHR2 Activators. **A** Nodes are colored and sized by the number of FRCs they respond to (defined as an FDR *p-value* < 0.05 when comparing chemical to DMSO control, no fold-change cutoff). Larger green nodes respond to a more chemicals, smaller brown nodes respond to fewer chemicals. General location of modules highly associated with FRCs (Modules 1, 2, 6, 7, and 10) are indicated. **B** Same as (**A**) but showing response to AHR2 Activators. Location of Module 13, highly associated with the AHR2 Activators, is indicated

To visualize how the network groups genes that respond to particular classes of chemicals, we overlaid gene expression changes from response to either the FRCs or the AHR2 Activators onto the full co-expression network. Overlaying gene expression changes was done by determining how many FRCs or AHR2 Activator chemicals each gene responded to (i.e. whether it was a DEG with *p*-value < 0.05 and log_2_FC > 1, for a given chemical treatment). Genes in the network were then sized and colored by how many chemicals they responded to with larger green nodes responding to more chemical treatments and smaller brown nodes responding to fewer chemical treatments. Genes responding to the FRCs were spread across several modules (Modules 2, 7, and 10, with some being in Modules 1 and 6, Fig. 3A). Functional enrichment showed that Module 1 was strongly enriched for development processes including multicellular, neuronal, and anatomical structure development. Module 2 was enriched for transport mechanisms including metal ion, cation and calcium transporters among others, similar to Module 6, which was enriched for sodium channel complexes (Table 1). In addition, Module 7 was enriched for regulation of non-motile cilium assembly, while Module 10 was enriched for axon development. The large number of FRC-responsive genes making up these modules strongly suggests that response to the FRCs in our dataset centers around these pathways and processes. In contrast to the FRCs, genes responding to the AHR2 Activators were very tightly localized to one location in the network (Module 13, Fig. 3B). Module 13 was specifically enriched for known chemical response pathways such as xenobiotic phase I and phase II metabolism, and oxidative stress, consistent with AHR functions [45, 46]. This module was also very tightly clustered (there were a number of edges linking genes within Module 13), indicating that the genes in this module are highly co-expressed relative to each other and are strongly regulated in response to AHR2 Activator exposure. Genes within Module 13 include known zebrafish AHR2-regulated genes upon PAH exposure including, *cyp1a, cyp1c1, ahr2, ahrra, ahrrb,* and *foxq1a* [39]. Thus, our chemical subgroup-specific module analysis shows that gene responses to FRCs are more spread across the network and are associated with several different pathways and developmental processes, while AHR2 Activators induce a much more focused and tightly controlled response consisting specifically of pathways linked to xenobiotic exposure. The remaining modules did not contain large numbers of genes that responded to either the FRCs or the AHR2 Activators and were likely driven by the remaining chemicals in our dataset. Several were enriched for general housekeeping processes in 48-hpf zebrafish [4], including neuronal (Module 5) and eye (Module 8) development (Table 1).

### Centrality analysis

While hierarchical clustering and module analysis can give broad overviews of pathway responses and modules associated with specific chemicals, networks also contain valuable gene-specific co-expression information. Centrality analysis of network genes based on betweenness or degree can reveal which genes are critical within the transcriptomic map of a biological system [34]. Genes of high degree are those with several connections to other genes and are termed “hubs”. Genes of high betweenness are those that occupy positions as links between larger clusters of genes, and are termed “bottlenecks”. Supplementary Figure S1A shows the top 20 genes from the network with the highest betweenness centrality values. The gene with the highest betweenness was *apc*, which codes for a regulator of the WNT signaling pathway [47] (Table 2). Bottleneck genes were also found throughout the network and in several different modules suggesting that such genes do not respond in a particularly strong way to any specific chemical or chemical class, but are associated either with general chemical response or with zebrafish housekeeping processes.

**Table 2:** Top 20 genes with highest betweenness in network

Gene	Function	Betweenness	Degree	Indegree^**a**^	Outdegree^**b**^	ModuleID
*apc*	APC regulator of WNT signaling pathway	0.27773144	9	3	6	1
*ubl3b*	ubiquitin-like 3b	0.24156245	7	2	5	1
*mapk7*	mitogen-activated protein kinase 7	0.2232999	5	4	1	1
*gata3*	GATA binding protein 3	0.18845384	8	3	5	1
*tfap2a*	transcription factor AP-2 alpha	0.07893617	11	4	7	1
*kdm5ba*	lysine (K)-specific demethylase 5Ba	0.07449596	3	1	2	1
*sf3b6*	splicing factor 3b, subunit 6	0.0737653	3	2	1	1
*pfklb*	phosphofructokinase, liver b	0.07243628	5	1	4	1
*kremen1*	kringle containing transmembrane protein 1	0.22835862	14	4	10	2
*cacna1da*	calcium channel, voltage-dependent, L type, alpha 1D subunit, a	0.10295178	30	5	25	2
*tfe3a*	transcription factor binding to IGHM enhancer 3a	0.07136245	30	11	19	2
*rusc1*	RUN and SH3 domain containing 1	0.06976945	6	2	4	3
*ppp2r3a*	protein phosphatase 2, regulatory subunit B″, alpha	0.25738053	4	2	2	4
*bhlhe23*	basic helix-loop-helix family, member e23	0.14505353	19	8	11	4
*fosl2*	fos-like antigen 2	0.10537935	18	7	11	4
*sgsm1a*	small G protein signaling modulator 1a	0.07037308	11	5	6	7
*ccdc43*	coiled-coil domain containing 43	0.09757468	3	2	1	9
*dimt1l*	DIM1 dimethyladenosine transferase 1-like (*S. cerevisiae*)	0.08821623	5	3	2	9
*g3bp1*	GTPase activating protein (SH3 domain) binding protein 1	0.07623317	7	4	3	11
*prr12a*	proline rich 12a	0.09610248	7	7	0	12

^a^ Number of edges emanating to the gene

^b^ Number of edges emanating from the gene

Genes of highest degree centrality (top 20) showed different location patterns within the network compared to genes of highest betweenness (Supplementary Figure S1B). Table 3 shows that the highest degree genes were primarily grouped into Module 13 (12 genes) which was enriched for AHR2 Activators, and Module 2 (7 genes), which was enriched for the FRCs. There was one additional high degree gene from the list of the top 20 that was found in Module 4. This degree centrality analysis shows that the most highly connected genes within our network are those specifically responding to AHR2 Activator or FRC exposure, in contrast to high betweenness genes which are more distributed throughout the network. The genes with the highest degree were *cyp1a* and *sult6b*, both previously shown to be highly induced by PAHs [39]. While the *cyps, gstp1*, and *ahrra* are all involved in response to xenobiotic stimulus and metabolism, the functional roles of the other high degree genes are less clear. *SlincR* and *foxq1a* appear to have roles in TCDD-induced toxicity in zebrafish; *slincR* was recently identified as a long noncoding RNA that is involved in the regulation of *sox9b*, one of the most highly depressed transcripts upon TCDD exposure [12], and *foxq1a* was induced by TCDD in the jaw primordium of developing zebrafish [13]. It is unknown what the specific roles of *gng13b, wfikkn1,* and *mxd1* are as they relate to chemical exposure; however, identification of these hub genes is suggestive of their potentially important roles in mediating toxicity of some of the chemicals in our network. We also highlight three high degree Module 2 genes that might be involved in pathways associated with disruption of neurodevelopment due to FRC exposure: *srgap3*, involved in neurodevelopmental processes as reviewed previously [48], *tfe3a*, an important transcription factor [49], and *cacna1da*, associated with calcium ion transport in neuronal signaling [50, 51]. Other high degree genes associated with FRC exposure are presented in Table 4, and the high centrality within their respective modules are suggestive of their potentially important roles in the toxicity of FRCs included in this study. We also examined which of these high degree genes responded to specific chemical treatments and found that many of them responded only to a subset of conditions (with *p-*value < 0.05, and log_2_FC > 1) (Supplementary Table S3). Therefore, while there was some overlap between centrality analysis and more fundamental pairwise comparisons to identify genes critical to chemical response, our network approach also found several new genes that would not have been found by simply comparing individual treatments to control. We also identified two genes of high degree that were completely uncharacterized, NA_732 (Module 13) and NA_146 (Module 2) (Entrez GeneIDs: 108182865 and 100,332,468 respectively). Identification of these uncharacterized genes in our analysis alongside genes that are strongly linked to AHR2 Activator or FRC exposure suggests that they should be investigated in future analyses.

**Table 3:** Top 20 genes with highest degree in network

Gene	Function	Betweenness	Degree	Indegree^**a**^	Outdegree^**b**^	ModuleID
*cyp1a*	cytochrome P450, family 1, subfamily A	0.00235481	41	17	24	13
*sult6b1*	sulfotransferase family, cytosolic, 6b, member 1	0.01272304	41	12	29	13
*cyp1c1*	cytochrome P450, family 1, subfamily C, polypeptide 1	0.00466897	39	17	22	13
*cyp1c2*	cytochrome P450, family 1, subfamily C, polypeptide 2	0.00712917	37	19	18	13
*gng13b*	guanine nucleotide binding protein (G protein), gamma 13b	0.03415074	37	12	25	4
*gstp1*	glutathione S-transferase pi 1	0.06511838	34	14	20	13
*wfikkn1*	WAP, follistatin/kazal, immunoglobulin, kunitz and netrin domain containing 1	0.00465263	34	16	18	13
*ahrra*	aryl-hydrocarbon receptor repressor a	0.0040985	33	17	16	13
*foxq1a*	forkhead box Q1a	0.00479027	33	13	20	13
*mxd1*	MAX dimerization protein 1	0.03254926	32	9	23	2
*slincR*	None	8.00E-04	32	15	17	13
*zgc.158689*	None	0.01270125	31	13	18	2
*cacna1da*	calcium channel, voltage-dependent, L type, alpha 1D subunit, a	0.10295178	30	5	25	2
*osbpl2a*	oxysterol binding protein-like 2a	0.01197251	30	8	22	2
*srgap3*	SLIT-ROBO Rho GTPase activating protein 3	0.01114073	30	10	20	2
*tfe3a*	transcription factor binding to IGHM enhancer 3a	0.07136245	30	11	19	2
*cyb5a*	cytochrome b5 type A (microsomal)	0.00566261	28	15	13	13
*pkhd1l1*	polycystic kidney and hepatic disease 1 (autosomal recessive)-like 1	0.04172331	28	12	16	13
*NA_732*	None	7.84E-04	27	13	14	13
*NA_146*	None	0.01266327	26	5	21	2

^a^ Number of edges emanating to the gene

^b^ Number of edges emanating from the gene

**Table 4:** Genes with the highest degrees from the modules associated with FRC exposure (Modules 2, 7, and 10)

Module	Degree	Gene	Name	Known function in zebrafish from previous studies	Reference
2	32	*mxd1*	Max Dimerization Protein 1	unknown	
	31	zgc.158689	unidentified	unknown	
	30	*osbpl2a*	oxysterol binding protein-like 2a	unknown	
	30	*srgap3*	Slit-Robo GTPase activating protein 3	role in neurodevelopmental processes	[46]
	30	*tfe3a*	Transcription factor-binding to IGHM enhancer 3a	Part of the MiT family coding for basic-helix-loop-helix/leucine zipper class transcription factors	[47]
	30	*cacna1da*	calcium channel, voltage-dependent, L type, alpha 1D subunit, a	calcium channel-related gene whose expression was shown to be altered by triadimefon, a broad-spectrum fungicide, and silica nanoparticle exposures to embryonic zebrafish	[48, 49]
7	18	NA_1170	uncharacterized		
	12	*purab*	Purine-rich element-binding protein Ab	unknown	
	11	*sgsm1a*	small G protein signaling modulator 1a	associated with small G protein-mediated signal transduction	[50]
	10	*fermt2*	fermitin family member 2	part of the Kindlin or Fermitin family of scaffold proteins important for signaling across membrane-spanning integrin adhesion receptors	[51]
10	10	*apc2*	APC regulator of WNT signaling pathway 2	in the family of genes coding for a regulator of the WNT signaling pathway	[45]
	10	*celsr3*	cadherin, EGF LAG seven-pass G-type receptor 3	plays a role in the facial motor neuron migration in zebrafish	[52]
	9	*adam22*	ADAM metallopeptidase domain 22	unknown	
	9	NA_108	unidentified	unknown	

### Analysis of module 13

**Fig. 4:**
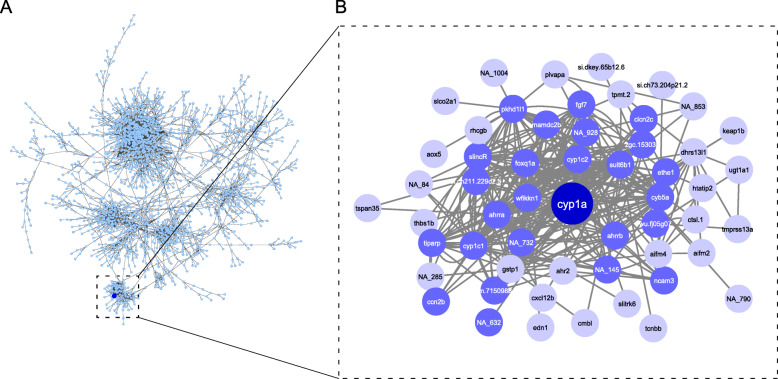
Network neighborhood of *cyp1a*. **A** The full network is shown with each blue circle (node) representing a zebrafish gene (2141 nodes are shown which represent the main network), and each line representing an edge (4373 edges are shown which represent the main network). The dark blue node represents *cyp1a*. **B** A subset network showing the network neighborhood of *cyp1a*. The *cyp1a* gene is in dark blue, nodes in blue represent those in the first-degree network neighborhood (directly linked to *cyp1a* through an edge). Nodes in light blue represent those in the second-degree network neighborhood (linked to *cyp1a* through one additional gene within the first network neighborhood)

Module 13 of the network is a critical AHR2 activation module associated with xenobiotic metabolism pathways. Centered within Module 13 is *cyp1a*, a well-studied biomarker gene of AHR exposure that is involved in detoxification of xenobiotics. This gene has been previously shown to be highly induced by TCDD and several PAHs [39, 52], has a high degree centrality in our network, and also responds to a large number of treatments (20/33 chemical treatments induce *cyp1a*). We extracted a subnetwork of the second order network neighborhood for *cyp1a* (Fig. 4A), which is defined as any gene connected directly to *cyp1a* through an edge, or any gene connected to a gene that is connected directly to *cyp1a*. Within this subnetwork, there were 55 genes (including *cyp1a*), and 339 edges. Several of the high degree Module 13 genes (Table 3) were present in *cyp1a*’s first degree network neighborhood including *sult6b1*, *cyp1c1*, *cyp1c2*, *gstp1*, *wfikkn1*, *foxq1a*, *ahrra*, and *slincR* (Fig. 4B). Additionally, *clcn2c*, *fgf7*, and *tiparp* were also part of this group. The second order network neighborhood of *cyp1a* consisted of *ahr2, gstp1*, and *dhrs13la*, and several other genes that have not been previously associated with PAH bioactivity in zebrafish. For genes in this group that have not yet been found to have a role in this response including *edn1*, *plvapa*, and *thsb1b*, and several uncharacterized genes, our analysis suggests that their roles as they relate to PAH exposure should be investigated in future studies.

**Fig. 5:**
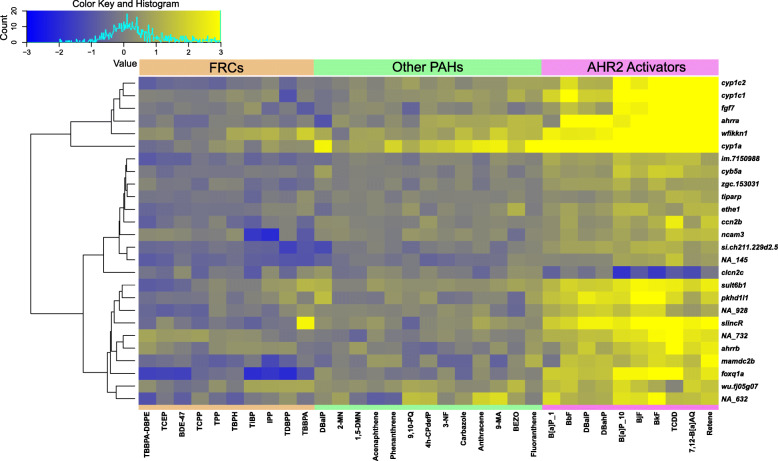
Heatmap of chemical response of genes in the first-degree network neighborhood of *cyp1a*. Genes are shown on the right (y-axis) and chemicals below (x-axis). Both genes and chemicals are clustered by similarity of response. Yellow indicates higher expression of each gene in chemical treatment compared to respective DMSO control, blue indicates lower expression in chemical treatment compared to respective DMSO control

We next examined the transcriptomic response of the first order network neighborhood genes of *cyp1a* to each chemical in our dataset. Figure 5 shows that while expression of *cyp1a* was increased with exposure to the AHR2 Activators, the expression of *cyp1a*’s network neighborhood genes showed some variability across all the chemicals, including the AHR2 Activators. Of note, even though *cyp1a* was induced by some of the other PAHs that were included in this study, the expression profiles of many of the first order network neighborhood genes were noticeably different from the AHR2 Activators (Fig. 5). Additionally, while most of the genes had increased expression with exposure to the AHR2 Activators, one exception was *clcn2*, which was decreased in its expression. However, among the other chemicals, the expression pattern of *clcn2* was similar to other genes in the *cyp1a* network neighborhood. Genes generally also showed a much stronger increase in response to the AHR2 Activators compared to their decrease in response to other chemicals. This was especially true for *cyp1a*, *cyp1c1,* and *cyp1c2*, showing that AHR2 Activators strongly induce increased expression of these genes. As with centrality analysis, we also compared how many chemical treatments each gene in the network neighborhood of *cyp1a* responded to (Supplementary Table S4). We found that many genes responded to only a few chemical treatments. Thus, their role in chemical response can be missed by fundamental treatment to control comparisons and only emerges when we take a network approach with a compendium of data as presented here. Interestingly, we also noted that of the 25 *cyp1a* network neighborhood genes, 21 responded to at least one FRC, with three genes (*foxq1a, NA_145,* and *si.ch211.229d2.5*) responding to at least six of the ten FRCs.

**Fig. 6:**
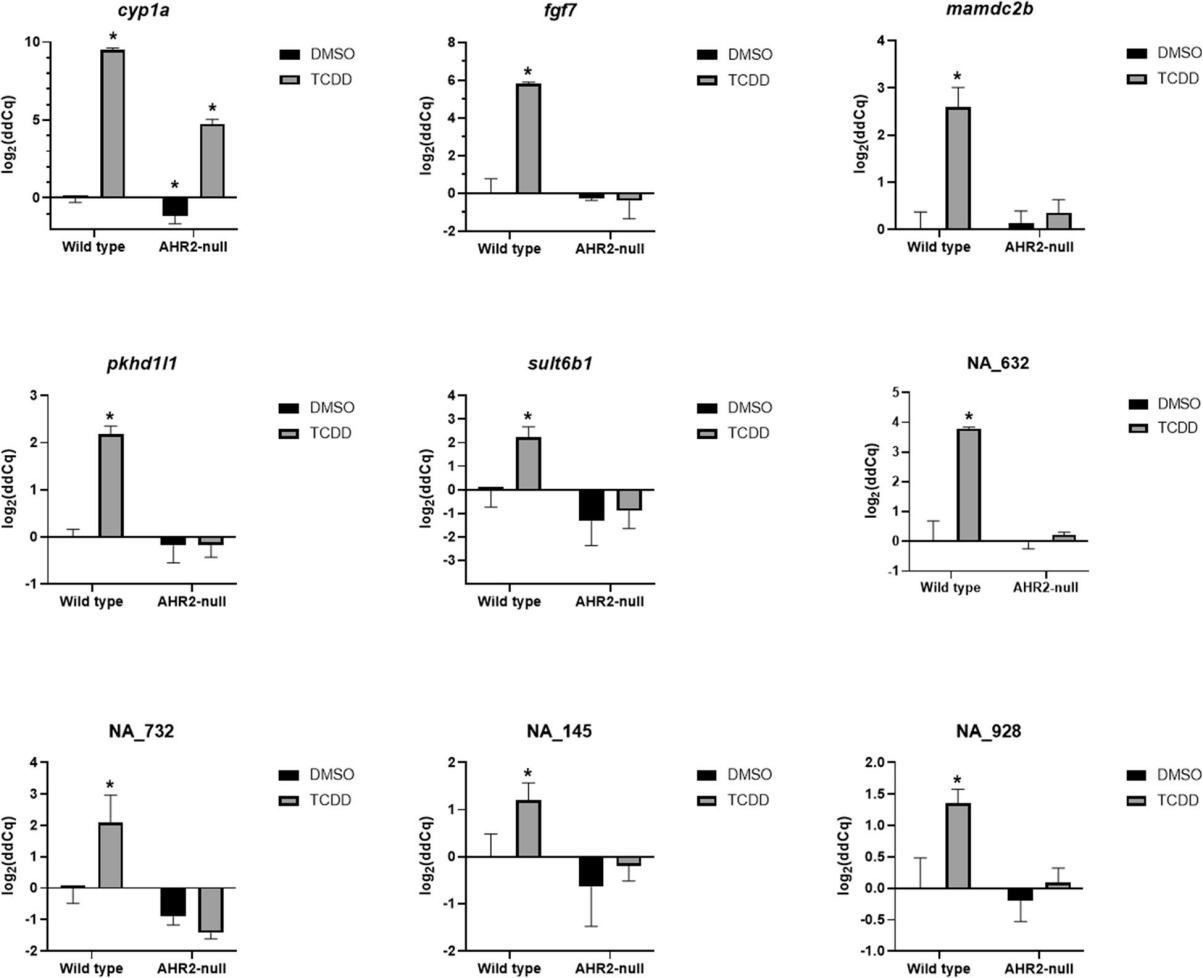
Validation of *cyp1a* network neighborhood genes identified from the network analysis. Comparative gene expression of *cyp1a* and selected *cyp1a* network neighborhood genes in 48-hpf wildtype and AHR2 mutant zebrafish developmentally exposed to 0.1% DMSO or 1 ng/mL TCDD (*n* = 3–4 biological replicates). Beta-actin was used as the normalization control. Error bars indicate SD of the mean. * = *p-value* < 0.05 compared to the wildtype vehicle control (DMSO). Statistical significance was determined using a Kruskal Wallis rank sum test followed by a Dunnett’s test for data that were not normal (NA_732), or a two-way ANOVA followed by a Tukey test for data that were normal (all other genes)

A combination of the presence in *cyp1a*’s network neighborhood (Fig. 4B) and the consistent induction of expression across all the AHR2 Activators (Fig. 5) led us to a subset of genes (*fgf7*, *mamdc2b, pkhd1l1, sult6b1*, NA_632, NA_732, NA_145, and NA_928) that we hypothesized would be closely associated with the AHR2 signaling pathway. To confirm the AHR2 dependence of these genes, we validated their expression upon TCDD exposure in both wildtype and AHR2-null zebrafish using qRT-PCR. We confirmed significant induction of all genes by TCDD, compared to vehicle-treated 48-hpf wildtype zebrafish (Fig. 6). Additionally, while *cyp1a* was significantly induced in TCDD-treated AHR2-null zebrafish, albeit significantly lower than TCDD-treated wildtype zebrafish, expression of none of the other transcripts changed in TCDD-treated AHR2-null zebrafish. These results confirm that our network analysis identifies multiple genes in the AHR2 signaling pathway.

### Chemical type analysis in networks

We next determined the relative contribution of chemical type towards the global functional pathways identified in our network by calculating how tightly network genes remain co-expressed when AHR2 Activator samples or FRC samples were removed from the dataset. This analysis reveals how genes of certain functional pathways are organized based on response to either the AHR2 Activators or the FRCs. When AHR2 Activator data was removed from our dataset and a new network was inferred, we found several functions whose constituent genes were less tightly co-expressed (based on their average co-expression values) when comparing to a network lacking random data (Table 5). This included negative regulation of vascular development, regeneration, and metabolic processes as well as carboxylic and oxoacid metabolic processes, and actin and cytoskeletal processes. The lower co-expression of genes within these functions when AHR2 Activators are removed indicates that they are particularly important to the transcriptomic response of AHR2 Activators. We also found functions whose constituent genes were more tightly co-expressed (suggesting lower importance to AHR2 Activators compared to all chemicals). This included bone, skeletal and cartilage development, as well as eye development.

**Table 5:**
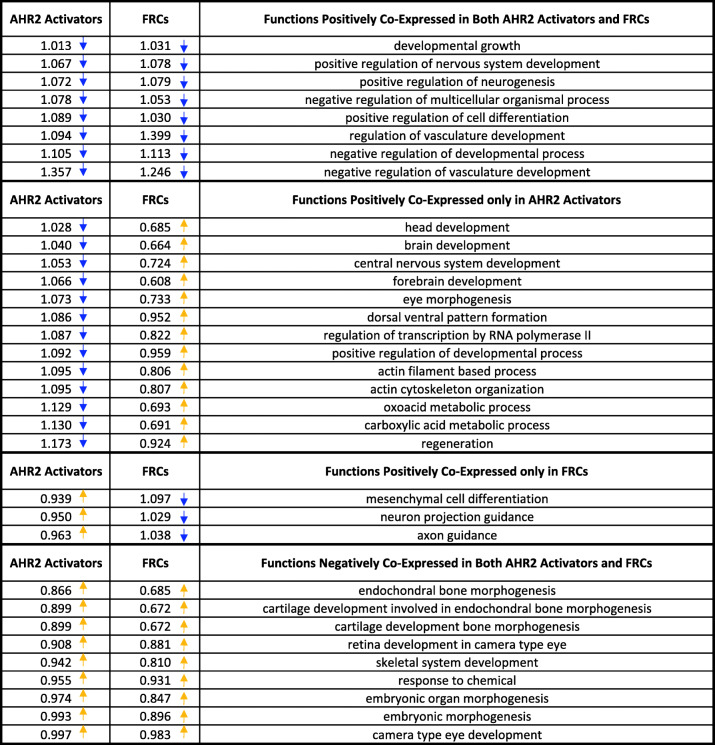
Removal of AHR2 Activators or FRCs from the network, and comparison of co-expression values of each pathway listed relative to networks lacking random datasets. The higher (yellow arrows) or lower (blue arrows) co-expression of genes within each of the listed functional enrichments in a network lacking AHR2 Activators (column 1) or FRCs (column 2) compared to a network lacking the same number of random chemicals as either the AHR2 Activators or FRCs, respectively

Functions showed even greater changes in co-expression when examining FRC data (Table 5). As above, FRC data was removed from our dataset and a new network was inferred and compared to a network lacking a random amount of data. The functions showing the greatest decrease in co-expression in a network lacking FRCs included regulation of vascular development and neurogenesis. Interestingly, many of the functions that showed higher co-expression were also related to neuronal development. These included brain, forebrain, head, and central nervous system development. This observation suggests that while vascular development and neurogenesis generally are pathways responding strongly to FRC exposure, the specific genes and sub-roles within these broad pathways may respond differently to FRC exposure.

## Discussion

The main goal of our study was to identify the important genes and biologically relevant pathways at the same zebrafish developmental timepoint that were associated with two groups of chemicals, the AHR2 Activators and the Flame Retardant Chemicals (FRCs), using a comparative network approach. We took advantage of a large compendium of 48-hpf RNA sequencing data from zebrafish exposed to 33 unique chemical treatments to conduct the first meta-analysis of zebrafish chemical transcriptomic data using gene co-expression networks. Using functional pathway enrichment paired with the systematic removal of the individual chemical groups from the network, we identified pathways associated with each of the chemical groups. The network revealed that the AHR2 Activators were associated with specific xenobiotic metabolism-related pathways while FRC exposure corresponded to broader, more general pathways related to perturbation of normal development, with an emphasis on neurogenesis.

While the genes in our full co-expression network formed into 23 distinct modules, genes responding to the FRCs or AHR2 Activators were found primarily in only three modules and a single module, respectively. Furthermore, while there was some overlap in modules associated with the two subsets of chemicals, most modules were associated with a certain chemical class suggesting largely distinct and highly specific molecular signaling events for these two chemical types. Our hierarchical clustering analysis reflected a similar pattern, where there was a strong separation between the FRCs and the rest of the chemicals in the study, but there was no separation between the AHR2 Activators and the remaining PAHs. Additionally, developmental toxicity phenotypes identified in previous studies were not a driver of our clustering. While high throughput screening for developmental toxicity is the necessary first step for determining perturbation to development [53], the toxicology field is now moving towards leveraging these data for mechanistic studies to reveal chemical modes of action. Thus, the network analysis here, showing that chemical types induce very specific transcriptional responses, provides a platform to characterize gene expression changes associated with a diverse group of chemicals and emphasizes one of the limitations of describing chemical toxicity with only phenotypic endpoints.

For our network analysis, we collected RNA sequencing datasets that had been previously published, and combined them with unpublished data gathered in this study. The resulting larger dataset has the advantage of examining gene expression changes associated with multiple chemicals at the same time point (48 hpf), and all at phenotypically anchored exposure concentrations. Such a dataset has never been analyzed before in zebrafish, allowing new conclusions to be gained here that describe transcriptomic response to chemicals. In addition to the strength of the dataset itself, we apply co-expression network analysis, an approach that is well designed for such a compendium of data. Network analyses can identify responses that lie outside the detection limit of more traditional pairwise comparisons of control and treatment conditions, and can also integrate data from a number of different studies and research groups [30, 54]. The variations in data collected across studies is an advantage for the network analysis we apply here as it represents additional biologically relevant variations in gene expression that can be used to infer edges between genes, leading to a more robust and accurate network. The combined strengths in this study (use of a comprehensive but coordinated dataset, and network analysis to move beyond traditional transcriptomic analysis) means that we were able to ask and answer questions that could not be queried in previous studies related to pathways responding to specific chemical treatments. This is reflected, for example, in our identification of high centrality genes that do not respond to specific chemical treatments but rather emerge only when the complete data set is analyzed with a network approach. The same is true for genes in Module 13; the close association of these genes, and the strong response of this module to AHR2 Activator exposure suggest that these genes are important to AHR2 activation. However, not all of the genes in Module 13 respond to all AHR2 Activators, and their association and likely role in chemical response only emerged when we applied network analysis to our large compendium of data. It is possible that the identification of Module 13 genes that do not respond to specific AHR2 Activators reflect the subtle differences in downstream transcription following AHR2 activation, and future work should investigate the functional roles of the identified gene expression changes upon exposure to each of the AHR2 Activator chemicals. Additionally, our observation that many of the Module 13 genes were also differentially expressed upon exposure to some of the FRCs (Supplementary Table S4) points to the fact that these genes might be involved in the general disruption to zebrafish development, probably via perturbation of AHR signaling. This is conceivable since the role of AHR in normal development has been identified previously across multiple species [55–57]. Indeed, it is true that some FRCs have previously been shown to activate AHR [26], although AHR activation might have been a result of contaminants present in the chemical standards [58].

The success of high-level analyses of large datasets has also been used in other studies. Schuttler, et al. used an approach based on building a map of transcriptomic response of genes and then clustering those that had similar profiles to view how genes were related [59]. Our approach here differs in that we use a network approach allowing for the application of much of the mathematical tools that have been developed for network analysis (centrality, etc) in addition to collecting a larger dataset. Li, et al. used transcriptomic data to better understand chemical response with a focus on cause and effect relationships, regulatory pathways, and cardiac responses specifically [60]. Aside from cardiac chemical response, network analysis has also been used to better understand heart regeneration in zebrafish [61]. Additional network analysis focused on Weighted gene co-expression network analysis (WGCNA) has also been applied to zebrafish, but again focusing on a smaller and more chemically homogenous dataset allowing for a detailed analysis of these chemicals and responses [62]. Our inclusion here of a large number of chemicals that show a range of phenotypic and transcriptomic responses lead a more comprehensive network that can highlight many different pathways and processes.

The compendium dataset we use here also has the advantage of examining different chemical classes with roughly the same level of analysis. However, despite the similar number of datasets included in the network analysis for each chemical subgroup, we found that FRC responses drove the majority of the network structure, while the AHR2 Activator gene expression changes were more restricted in the network, which was likely due to the diversity of structures and molecular initiating events of the FRCs compared to the AHR2 Activators. Both functional enrichment of FRC-responsive modules, as well as analysis of functions shifting their co-expression after removal of FRC datasets show that the FRCs influence functions related to developmental, neurological, and signaling and transport pathways. The analysis removing the FRCs from the network identified regulation of vascular development as being particularly important to this group of chemicals. Vascular developmental abnormalities and cardiac arrhythmia have been investigated in zebrafish exposed to only few FRCs [27, 63, 64]; but our results highlight it as a critical organ system that should be more thoroughly investigated when studying FRC toxicity. Our conclusions also corroborate evidence from a number of previous studies demonstrating zebrafish FRC neurobehavioral toxicity upon exposure to multiple structurally diverse chemicals, including individual organophosphate FRCs [22, 65], DE-71 (a PBDE) [66], and TBBPA [67]. Additionally, all FRCs included in this network analysis were previously determined to induce both 24-hpf and 120-hpf behavior effects in zebrafish, albeit at varying degrees [6]. Thus, our network analysis not only confirmed observations from previous studies, but also provides guidance for future studies investigating mechanisms of FRC developmental toxicity. We recognize that even though the FRCs as a group lit up distinct parts of the network compared to the rest of the chemicals in the study, the structurally distinct chemicals within the FRCs are likely to have some unique modes of toxicity that were not captured here. Our analysis contained only a limited number of FRCs from each structural class (for example, there were two brominated phenols, TBBPA and TBBPA-DBPE), and future work should consider incorporating more FRC transcriptomic data, and using this increased FRC dataset to view how different types of FRCs induce gene modules or functional pathways. Our understanding of the mechanisms by which the structurally diverse FRCs cause developmental toxicity is still in its infancy. This is likely because several FRCs have been shown to interact with multiple nuclear receptor signaling pathways to cause gene expression changes and developmental toxicity in zebrafish [68, 69]. Thus, our discovery of some of the high centrality genes in our network such as *kremen1, cacna1da,* and *tfe3a* within Module 2 (highly associated with the FRCs), emphasizes their potentially important role in driving FRC transcriptomic responses. Tfe3 is a transcription factor, and its high co-expression with genes associated with FRCs suggests that it might be involved in regulation of the genes within this module. The identification of the other high centrality genes present in the FRC modules (Table 4) should be investigated in future research to understand their roles in the pathways enriched by their modules.

Module 13, whose genes were enriched for xenobiotic metabolism by cytochrome P450, had a prominent role in our network analysis. Although Module 13 was a small module, it was very tightly co-expressed, and contained a large number of high degree genes. This is in line with our previous studies showing that high degree genes are those that are heavily involved in a small number of specific pathways [70]. This is in contrast to high betweenness genes which are often associated with several pathways but not strongly with any one pathway, in agreement with their general position linking multiple larger groups of genes. Previous studies in centrality have found both degree and betweenness to be approximately equal in their identification of genes of importance [34]. However, those studies looked at a broader range of conditions than we do here, where we infer a network very focused on chemical response. This may suggest that in more focused networks inferred from very similar datasets using degree as the primary centrality measure may be advantageous, though further studies are needed to determine if this is an effect of network structure generally or of the network presented here in particular. However, it does suggest that for this specific network when identifying genes that respond to certain chemicals of interest, degree is a better metric of importance than betweenness.

Module 13 was primarily made up of genes responding to the AHR2 Activators, which consisted of TCDD and several PAHs, and at the center of this module was the *cyp1a* gene, a widely use biomarker for PAH exposure in several organisms [71, 72]. We found several AHR signaling pathway genes such as *ahrra* and *ahrrb* [73], *foxq1a* [13]*,* and *slincR* [12] within Module 13 that were tightly co-expressed with *cyp1a.* More interestingly, our network analysis also identified within Module 13 other annotated and non-annotated genes, including novel long non-coding RNAs that have not been previously associated with AHR2. We hypothesized that they too were involved in the AHR signaling pathway. Our in silico network analysis conclusions are corroborated through our RT-qPCR analysis demonstrating that *fgf7*, *mamdc2b*, *pkhd1l1*, and *sult6b1* expression is dependent on the presence of AHR2. Importantly, several novel genes, NA_632, NA_732, NA_145, and NA_928 (Entrez GeneIDs of 103,910,027, 108,182,865, 100,332,446 and 407,643 respectively) were also associated with the AHR2 signaling pathway (Fig. 6). NA_732 and NA_928 are long non-coding RNAs, while NA_632 and NA_145 are protein coding genes. Despite that fact that the functions of these genes are unknown, their presence in Module 13 linked to *cyp1a* strongly suggest that they are important to AHR2 signaling. Future functional studies investigating the roles of these novel AHR2-related genes will help strengthen our mechanistic understanding of AHR2 Activator toxicity.

Overall, the methods used in this study combined the strength of using an extensive transcriptomic dataset with a gene co-expression analysis approach to measure how a diverse group of chemicals altered the 48-hpf zebrafish transcriptome relative to each other. We found that the FRCs and AHR2 Activators localized to distinct regions of the network we created, highlighting very specific transcriptomic responses to each chemical group. Additionally, FRCs induced a broad response related to neurobehavior, ion signaling, and vascular development, while the AHR2 Activators centered in one module related to chemical stress and metabolism-related responses. Guided by our network, we also discovered novel genes associated with the AHR2 signaling pathway. Overall, while the FRCs and AHR2 Activators have chemical-specific gene expression changes, we also identified several candidate biomarker genes that future studies should focus on to gain a better understanding of the toxicity of these two chemical groups. While the toxicology field has thus far focused on understanding phenotypic responses associated with chemical exposure, we are now transitioning to unraveling the mechanisms of chemical hazard, which will enable more in-depth characterization of chemicals. This study provides transcriptomic biomarkers that could be used in the future for exposure determination and mixture component diagnosis as they relate to mode-of-action based risk assessment of PAHs. Additionally, the transcriptomic network that we created can be used as a resource for future studies investigating mechanisms of toxicity in developing zebrafish.

## Methods

### Characterization of chemical datasets

In total, we collected RNA sequencing data from 48-hpf zebrafish exposed to 33 unique chemical treatments (Supplementary Table S1). For 29 of these treatments (18 PAHs, TCDD, and 10 FRCs), the data was collected from previously published previous studies [7, 39–41], with the remaining 4 treatments (4 PAHs) initially analyzed in this study. Each treatment was examined with 3–8 replicates for a total of 170 RNA sequencing samples included in the study. Raw and processed RNA sequencing files for each sample have been deposited in NCBI’s GEO database (GSE171944). See Supplementary Table S1 for information on chemicals, exposure levels, and relevant references for the datasets used in this study.

#### Chemicals

Detailed methods for published datasets can be found in our previous studies [7, 39–41]. Methods for chemicals initially analyzed in this study, and TCDD exposures for RT-qPCR analysis (See below) are described here. The PAHs (benzo [a] pyrene (B [a]P), 9,10 phenanthrenequinone (9,10-PQ), and dibenzo [a,l] pyrene (DBalP)) were dissolved to 10 mM in 100% DMSO and stored in a desiccator. TCDD was purchased from Supelco (Sigma Aldrich) at 311 nM with 95.3% purity, and stored in the dark at room temperature. The chemical stocks were sonicated in a water bath sonicator for 15 min (PAHs) or 30 min (TCDD).

#### Zebrafish husbandry

The study’s zebrafish protocols were performed according to the relevant guidelines provided by the Oregon State University’s Institutional Animal Care and Use Committee protocols (ACUP 5143). Briefly, Tropical 5D wildtype zebrafish were housed at Oregon State University’s Sinnhuber Aquatic Research Laboratory (SARL, Corvallis, OR) in densities of 1000 fish per 100-gal tank. Fish were maintained at 28 °C on a 14:10 h light/dark cycle in recirculating filtered water, supplemented with Instant Ocean salts. Adult fish were fed GEMMA Micro 300 or 500 twice a day, and larval and juvenile fish were fed GEMMA Micro 75 and 150, respectively, three times a day [74]. Spawning funnels were placed in the tanks at night and the following morning embryos were collected and age staged [4, 75]. Embryos were maintained in embryo medium (EM) in an incubator at 28 °C until further processing. EM consisted of 15 mM NaCl, 0.5 mM KCl, 1 mMMgSO4, 0.15 mMKH2PO4, 0.05 mM Na2HPO4, and 0.7 mMNaHCO3.

#### Exposures and RNA-sequencing sample preparation and sequencing

Detailed exposure methodology and phenotypic endpoints identified for each chemical for the previously published datasets can be found in their respective studies [7, 39–41]. Briefly, for the PAHs (with the exception of 7,12-B [a] AQ and BEZO) and FRCs, an EC80 exposure concentration was selected based on a concentration-response zebrafish phenotypic screen [6, 38]. If an EC80 concentration was not attainable, the maximum concentration tested during the phenotypic screen (50 μM for PAHs, and 85 μM for the FRCs) was utilized for RNA sequencing [39, 41]. 7,12-B [a] AQ and BEZO were exposed at their EC100 concentrations [7], and 1 ng/mL TCDD was utilized, which is a concentration that ensures 99–100% of zebrafish at 120 hpf have the expected TCDD-induced toxicity endpoints [40]. All samples were processed for RNA sequencing analysis at 48 hpf, a timepoint whose transcriptome precedes and likely drives morphological and/or behavioral phenotypes observed at 120 hpf [7, 39–41]. Fastqc files for each chemical were re-analyzed in this study (See “Alignment and analysis of RNA-seq data” below).

For the PAHs (B [a] P, 9,10-PQ, and DBalP) initially analyzed in this study, zebrafish embryos were dechorionated using pronase at 4 hpf, and were batch-exposed to chemicals at 6 hpf in glass vials as described previously [7]. Exposure concentrations were 1 and 10 μM for B [a] P, 1.2 μM for 9,10-PQ, and 10 μM for DBalP. The vehicle control was 1% DMSO, and there were 20 embryos per glass vial in 2 mL exposure solution. Vials were incubated at 28 °C in the dark on a rocker until sample collection at 48 hpf. Following exposure, 48-hpf whole embryos were homogenized using RNAzol (Molecular Research Center, Inc.) and a bullet blender with 0.5 mM zirconium oxide beads (Next Advance), as recommended by the Next Advance. Each biological sample consisted of 20 pooled 48-hpf zebrafish. The RNA from the PAH exposures was isolated via phenol guanidine extraction. RNA integrity was assessed (RIN score > 9) using an Agilent Bioanalyzer. Total RNA samples were sent to the Oregon State University Center for Genome Research and Biocomputing Core facilities for library preparation and sequencing. This included mRNA enrichment by polyA selection. Libraries were prepared with the PrepX™ mRNA and Illumina sequencing workflow (Wafergen Biosystems). 50 bp paired-end sequencing was conducted using an Illumina HiSeq 2000 sequencer.

### Alignment and analysis of RNA-seq data

All of the datasets listed in Supplementary Table S1 were analyzed (or re-analyzed if already published) using the following approach. Each fastq file was aligned to the Genome Reference Consortium Zebrafish Build 11 (GRCz11) (https://www.ncbi.nlm.nih.gov/assembly/GCF_000002035.6/) using the Star Aligner [76] with default settings. Resulting SAM files were then used to count reads aligning to genes using HTSeq (https://htseq.readthedocs.io/en/release_0.11.1/) [77] along with the gff file for the GRCz11 genome, resulting in raw read counts for 39,701 zebrafish genes. Low expression genes, defined as those with a count of ‘0’ in at least 43/170 (25%) of samples, were removed from further analysis. The final raw dataset consisted of 21,854 genes across 170 samples.

Raw counts were normalized using Bioconductor’s DESeq2 package [78]. DESeq2 was also used to calculate log_2_ fold changes (log_2_FC) and adjusted *p*-values (corrected for multiple hypothesis testing) for the 33 individual chemical comparisons to their respective DMSO controls. Adjusted *p*-values and log2FC values for all 21,854 genes are shown in Supplementary Table S5. In addition, whether the gene is a DEG (log2FC > 1 and an adjusted *p*-value < 0.05) is indicated. To further reduce the number of genes used to infer a network, any gene that was not differentially expressed (defined as an adjusted *p*-value > 0.05, no fold change cutoff) in at least 3 of the comparisons (10% of the total) was removed from further analysis. This resulted in 10,346 genes across 170 samples from 33 conditions being included in subsequent networks. Log_2_FC values (comparing chemicals to their respective DMSO controls) of these 10,346 genes were then used to infer the gene co-expression network.

### Inferring gene co-expression networks

GENIE3 [31] was used to generate a matrix of co-expression values for all gene pairs. Regulators or targets were not pre-selected so as to allow all gene co-expressions to emerge from the analysis. The tree method was randomforest, and the number of candidate regulators randomly selected at each tree node (for the determination of the best split) was set to the square root of the total number of candidate regulators. The number of trees in an ensemble for each target gene was set to 1000. All of these settings represent the default values for GENIE3. We filtered low co-expression values (those below 0.00858) to generate a network with sufficient structure for topological analysis, as done previously [70, 79]. Networks were viewed in Cytoscape [80] by importing a .sif file with each line indicating a gene pair connected by an edge. A prefuse force directed layout was used to view the network. We also used Cytoscape to calculate centrality values by using the Analyze Network tool and analyzing as a directed network. Network neighborhoods of genes of interested were identified by selecting a gene of interest and using Cytoscape to highlight the first network neighborhood of the selected gene. This was followed by highlighting the first network neighborhood of each of the selected genes (corresponding to the second network neighborhood of the original selected gene). The 23 modules in the network were determined using the fastgreedy.community function within the igraph package in R [81] with the minimum number of genes comprising a module set to 12. Functional enrichment of modules was done using g:Profiler using default settings [44]. A function was considered to be enriched if the *p*-value of the enrichment was below 0.05, and the function was overrepresented in a module or network compared to the zebrafish genome as a whole. Underrepresented functions were not considered. g:Profiler outputs functions from KEGG, Gene Ontology, Reactome, and WikiPathways. We examined all enriched functions regardless of which database they were drawn from. This led to some overlap in enriched functions but also allowed us to gain the most comprehensive view of pathways enriched in certain parts of the network or in different networks.

To determine which functions and pathways may be related to certain classes of chemicals, we selected two subsets of our data, chemicals in the FRC class or chemicals in the AHR2 Activator class (Supplementary Table S1). For FRC analysis, we first inferred a GENIE3 co-expression matrix after removing FRC data (comprising 39 samples). Next, we randomly removed 39 samples of data from our dataset and calculated the resulting gene co-expression matrix. This random data removal from the full dataset and matrix calculation was repeated an additional nine times and an average gene co-expression matrix was calculated from these ten iterations of data removal. A network was then constructed from this average gene co-expression matrix. This network represents the effect of randomly removing data from our constituent dataset. This random removal network was then compared to the network inferred after specifically removing FRC data. For each of these two networks (that lack FRC data or lack an identical amount of random data), we calculated the average co-expression value between all genes that belonged to each functional enrichment identified using g:Profiler [44]. Genes of a particular function that show lower co-expression in a network that is specifically lacking FRC data compared to a network that has data randomly removed from it, suggests that this function is especially critical to the FRC response. A similar pair of networks and comparisons were made for the AHR2 Activators (comprising 46 samples).

### TCDD exposure, RNA extraction, and quantitative reverse transcriptase polymerase chain reaction (qRT-PCR)

To confirm that the highly co-expressed genes associated with the AHR2 Activators in our network are in the AHR signaling pathway, we performed a qRT-PCR experiment. Wildtype and AHR2-null [56] zebrafish embryos were exposed to 0.1% DMSO or 1 ng/mL TCDD at 6 hpf, as described previously [40], in a manner similar to the TCDD RNA sequencing samples included in this study. The 1 ng/mL TCDD concentration was selected as it results in 99–100% of 120-hpf zebrafish displaying the expected TCDD-induced phenotypic malformations such as heart and cartilage malformations [82]. Briefly, exposures were conducted in 20-mL amber glass vials with 1 embryo/100 μL exposure solution for 1 h with gentle rocking. Vials were also inverted every 15 min to ensure even exposure. After exposure, embryos were rinsed three times with EM, transferred to 100-mm Petri dishes, and raised in EM at 28 °C to 48 hpf. RNA was extracted in a manner similar to the PAHs, as described above. RNA quantification and quality assessment (O.D. 260/280 ratios) was conducted using a BioTek® Synergy™ Mx microplate reader with the Gen5™ Take3™ module.

qRT-PCR was conducted in 10 μL reactions consisting of 5 μL SYBR® Green Master Mix, 0.08 μL reverse transcriptase enzyme mix (Power SYBR® Green RNA-to-CT™ 1-Step Kit; Applied Biosystems), 0.2 μL each of 10 μM forward and reverse primers, and 20 ng RNA per reaction. The gene specific primers (IDT) for qRT-PCR amplification are listed in Supplementary Table S6. The QuantStudio 5 Real-Time PCR System (Thermo Fisher Scientific) was used and the cycling parameters were as follows: reverse transcription at 48 °C for 30 min, denaturation and activation of SYBR® polymerase at 95 °C for 10 min, followed by 40 cycles of amplification (95 °C for 15 s, 60 °C for 1 min). A melt curve analysis was conducted to assess for multiple products, and it was confirmed that all primers amplified a single product. Expression values were normalized to the β-actin control, and analyzed with the 2^−ΔΔCT^ method as described previously [83]. The data collected for each gene was tested for normality using the Shapiro-Wilk normality test. To determine significant difference compared to control (*p-value* < 0.05), either a two-way ANOVA with the post-hoc Tukey’s test or a Kruskal Wallis rank sum test followed by a Dunnett’s test was conducted depending on if the data that passed or failed the normality test, respectively,. Data analysis was conducted using RStudio (version 3.6.0), and data visualization was performed using GraphPad Prism version 8.0.0 for Windows, GraphPad Software, San Diego, California, USA (www.graphpad.com).

## Supplementary Information

**Additional file 1.**

**Additional file 2.**

## Data Availability

The raw and processed files for all datasets used in this study are in NCBI’s Gene Expression Omnibus (GEO) database (Accession number: GSE171944).
