# Supplementary material for: Gene co-expression network analysis in zebrafish reveals chemical class specific modules
Source: BMC Genomics. 2021 Sep 13;22:658. doi: 10.1186/s12864-021-07940-4 (PMC8438978; doi:10.1186/s12864-021-07940-4)
Supplement: Supplementary file 2 — Additional file 2. [file 12864_2021_7940_MOESM2_ESM.docx]

**Gene Co-expression Network Analysis in Zebrafish Reveals Chemical Class Specific Modules**

Prarthana Shankar^‡^^1^, Ryan S. McClure^‡2^, Katrina M. Waters^2^, Robyn L. Tanguay^1*^

*^1^ Department of Environmental and Molecular Toxicology, Oregon State University, Corvallis, OR 97331 USA*

*^2^ Biological Sciences Division, Pacific Northwest Laboratory, 902 Battelle Boulevard, P.O. Box 999, Richland, WA 99352 USA*

‡ These co-authors contributed equally to this manuscript.

* Correspondence to:

Robyn Tanguay, Ph.D. Department of Environmental and Molecular Toxicology, the Sinnhuber Aquatic Research Laboratory 28645 East Highway 34, Oregon State University, Corvallis, OR 97333. Email: Robyn.Tanguay@oregonstate.edu, Telephone: 1-541-737-6514, Fax: 1-541-737-6074

**Supplementary figures**


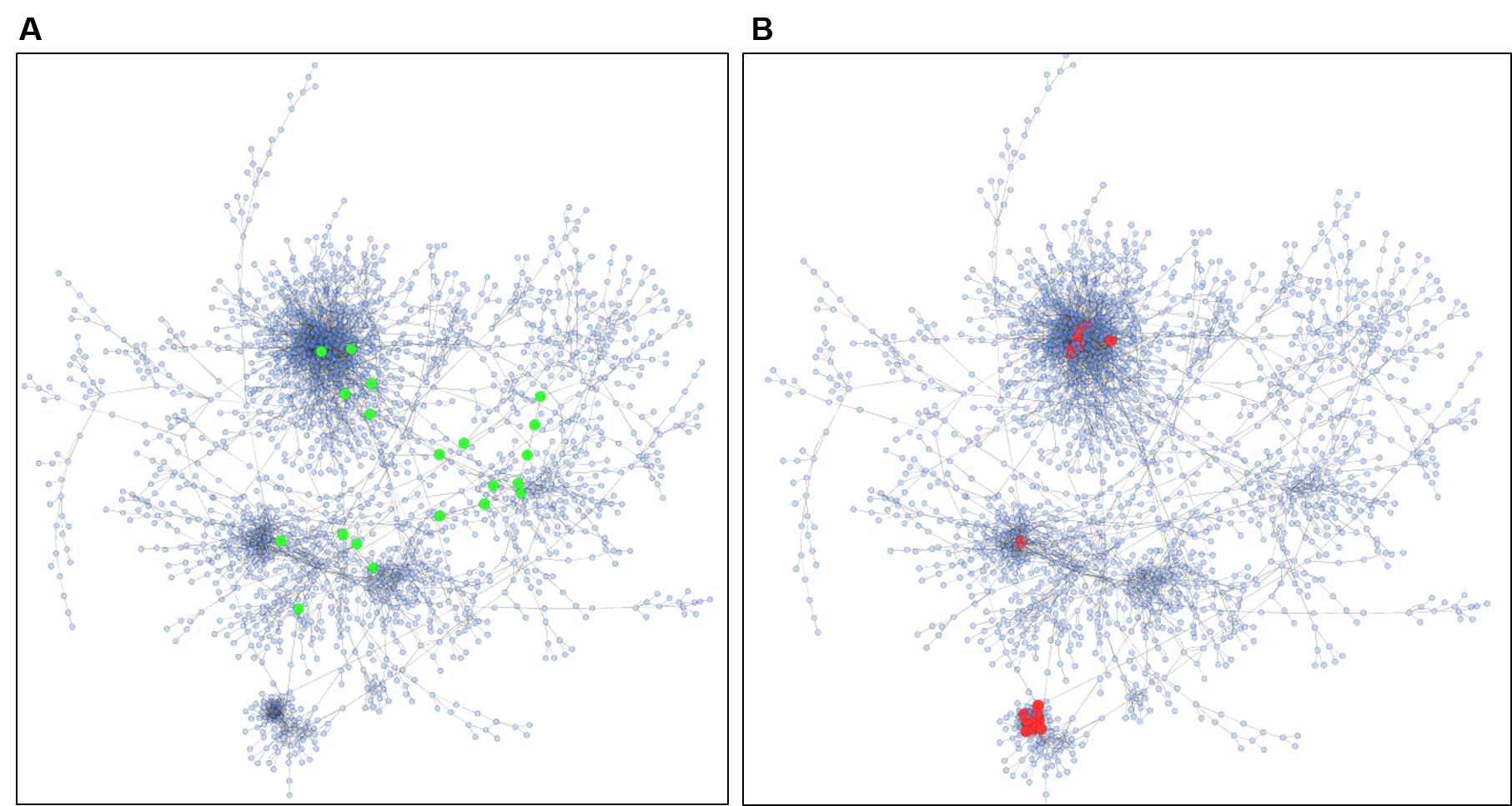


**Figure S1. High Centrality Network Genes. (A)** Genes of high betweenness (top 20 ranked by betweenness) are indicated as larger green nodes in the network. **(B)** Genes of high degree (top 20 ranked by degree) are indicated as larger red nodes in the network.
